# ﻿An updated Atlas of *Helophorus* chromosomes

**DOI:** 10.3897/compcytogen.17.112831

**Published:** 2023-12-21

**Authors:** Robert B. Angus

**Affiliations:** 1 Department of Life Sciences (Insects), The Natural History Museum, London SW7 5 BD, UK The Natural History Museum London United Kingdom

**Keywords:** banding, chromosomes, experimental hybrids, *Helophorus*, karyotypes, parthenogenesis, triploidy

## Abstract

An account is given of my development of techniques to obtain well-spread Giemsa-stained banded chromosome preparations. Apparent G-banding could be obtained following very slight trypsin treatment of freshly prepared slides, but this banding was very fine (close-grained) and possibly not a reflection of chromosome structure. However, treatment of developing embryos *in vitro* with 5-fluorouridine produced a similar chromomere banding, which is therefore regarded as genuine. Steady accumulation of *Helophorus* Fabricius, 1775 karyotypes has resulted in the production of an Atlas covering 62 of the 170 species known to occur in the Palaearctic. Chromosome polymorphisms involving pericentric inversions and addition of extra C-banding regions have been found, as well as small B-chromosomes in a few species. In general, karyotypes have proved very useful in establishing the limits of individual species. Parthenogenesis involving triploidy has been found in two species. Karyotypes of experimentally produced hybrids have revealed irregularities in chromosome condensation.

## ﻿Introduction

My investigation of *Helophorus* chromosomes began in 1975 with my appointment as a Lecturer in the Zoology Department of Royal Holloway College, University of London. Earlier attempts at chromosome preparation had resulted in complete failure, but now the field was beginning to open up. The paper by Crozier (1968) describing an acetic acid dissociation, air-drying technique was a breakthrough. It allowed preparations of well spread undistorted chromosomes. Initially Crozier had used aceto-lactic orcein staining, but application of Giemsa stains had already been described for similarly prepared mammalian chromosomes (Rothfels and Siminovitch 1958) and this gave excellent results. All my early preparations were from developing embryos.

In those early days insect chromosomes were known to display C-banding and to show active nucleolus organisers (NORs) by silver staining. G-banding was another matter.

C-banding is associated with highly repetitive DNA, with one base-pair to a short sequence of base-pairs repeated many times. Such bands are present in both dividing and interphase chromosomes. It is generally observed following treatment with alkali (for me saturated Ba(OH)_2_ at room temperature), followed by incubation in salt-sodium citrate (2X SSC) at about 60°C. There have been attempts to differentiate “true C-bands” from other less distinctive types. With beetles a pretreatment with 1N HCl has been recommended–applied to my chromosome preparations it abolishes all traces of banding!

I have found silver staining tricky. I have not succeeded with acetic acid inflated material but can get it to work with centrifuge-spread material. The results are consistent.

G-banding is where the real rewards may lie, enabling chromosomes and even sections of chromosomes to be identified with great precision, demonstrating homologies between chromosomes of different species and their relatedness as with Man and the Great Apes (Pearson 1997) and the Giant Panda and the Brown Bear (O’Brien et al. 1985).

Published information on G-banding was not encouraging. Maudlin (1974) published information on G-banding in triatomine bugs (Heteroptera), but in most of the chromosomes there are only a few bands. Steiniger and Mukherjee (1975) obtained banding patterns in the mosquito *Aedesalbopictus* (Skuse, 1895) by reducing normal fixation times. The results appear dramatic but ragged and certainly not fine-grained. Webb (1976) obtained spectacular banding on B-chromosomes of the Australian plague locust *Chortoicetesterminifera* (Walker, 1870). These B-chromosomes are heterochromatic and the bands were in the same positions whether resulting from G- or C-banding protocols. Tambasco et al. (1974) reported G-banding in South American stingless bees, but again the bands were few in number, and hard to see in the photograph.

**Figure 1.**
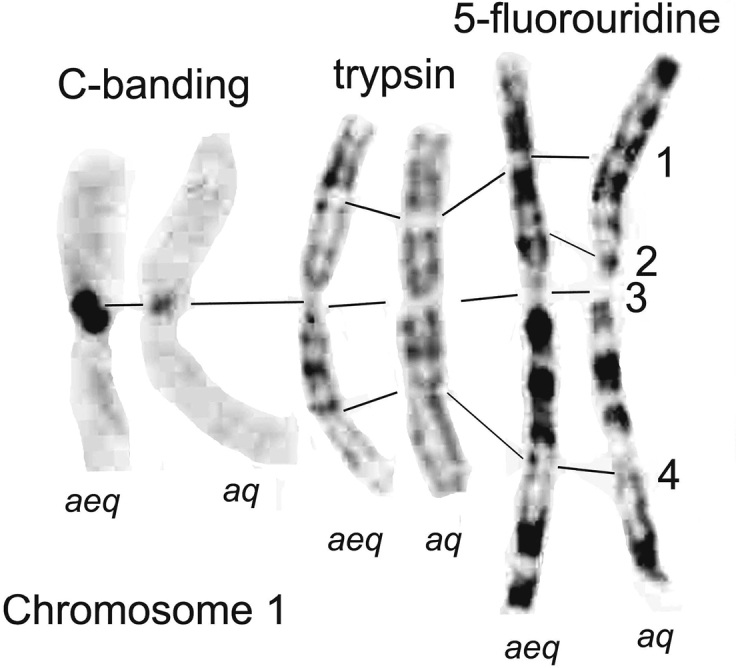
Detailed comparison of the banding patterns of Chromosome 1 of *Helophorusaequalis* (*aeq*) and *H.aquaticus* (*aq*). The lines joining the chromosomes indicate homologous points. Treatments are indicated above the illustrated chromosomes.

Bigger (1975), using the centrifugation method with cell suspensions, produced what he claimed to be G-bands on various Lepidoptera including the Large White butterfly, *Pierisbrassicae* (Linnaeus, 1758). His published photographs are difficult to interpret, and his diagrams are interesting but may have to some extent been guided by the “eye of faith”. However, Dutrillaux et al. (2022), using more refined microscopy, especially confocal microscopy, showed localized primary constrictions as well as some banding. In 2004, working with L. A. Dutton, then an undergraduate student doing a research project, I obtained some well-spread preparations from eggs, treated with 5-fluorouridine. Although not nearly as convincing as the *Helophorus* chromosomes shown in in Fig. 1 of this paper, they did hint at possible fine-grained banding.

After much experimentation I found that bands could be produced by a very slight trypsin treatment of freshly prepared slides (5 min. drying immediately after preparation)–0.01% Difco 1:250 trypsin in 0.75% NaCl buffered to pH 7.6 with Sörensen - for 5–15 sec. at 10°C, then quenched by rinsing in three changes of distilled water at pH 6.0. This is a very slight treatment but can give very good results, with numerous bands on all the chromosomes, inviting the hope that results comparable with those obtained from bear chromosomes might be possible. The problem was, the banding produced is not only very fine-grained but also very even, so did it reflect chromosome organisation or merely the last bits not destroyed by the trypsin? A solution came from studies by Rønne and others using various “antibiotic” reagents on *in vitro* cultures of human cells (Rønne 1977); (Rønne and Andersen 1978). Cycloheximide, chosen because it was relatively cheap, had very limited success, but 5-fluorouridine, which appeared to give the clearest results with human cells (Rønne and Andersen 1978) gave some very clear results (Fig. 1). Fig. 1 shows a comparison of the longest autosomes of *Helophorusaquaticus* (Linnaeus, 1758) and *H.aequalis* Thomson, 1868. The chromosomes of the two species show a similar sequence of bands (allowing for the different sizes of their centromeric C-bands), except for the distal region of the short arm, beyond a fairly distinct gap in about the middle of the arm. In *H.aquaticus* there are three very distinct bands in this distal section, but in *H.aequalis* the bands are less distinct, comprising a basal one with a hint of subdivision and apical less stained and more indistinct section. The conclusions are that the banding reflects the chromomeric organisation of the chromosomes, and that the apical sections of the short arms have been involved in translocations. The details are explored further by Angus (1982).

The extent to which this fine-grained banding, however useful, is the same as the G-banding obtained with mammalian chromosomes remains to some extent an open question. One interesting feature of mammalian G-banding is that the bands correspond with those observed on pachytene chromosomes during meiosis (Luciani et al. 1975). Dutrillaux et al. (2006) developed methods of using pachytene banding in beetles, and some of these bands appear very similar to the fine-grained banding in *Helophorus*. It therefore seems that these are G-bands.

**Figure 2.**
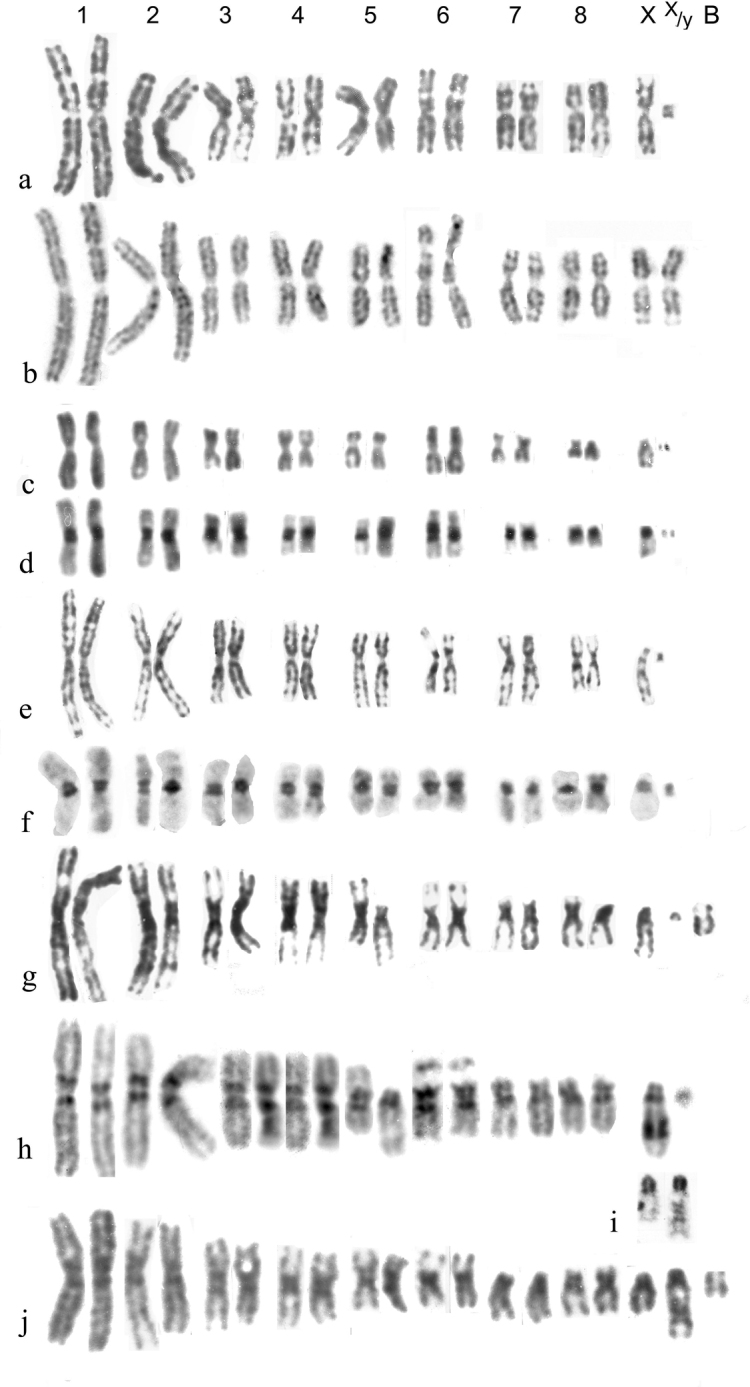
**a–j***Helophorus* str. Mitotic chromosomes arranged as karyotypes **a, b***H.aquaticus*, embryos, banded with trypsin **a** ♂, France, Fontanières **b** ♀, Russia, Strelna near St Petersburg **c, d***H.thauma*, paratype ♂, mid gut **c** Giemsa-stained **d** the same nucleus, C-banded **e, f***H.aequalis*, France, mid gut **e** Giemsa-stained **f** C-banded **g–j***H.grandis*, embryos **g** ♂, France, Giemsa-stained **h** ♂, Russia, Pavlovsk near St Petersburg, C-banded **i** long and short X chromosomes C-banded by silver-staining **j** ♀, England, Surrey showing the long and short X chromosomes. Scale bar: 15 µm.

**Figure 3.**
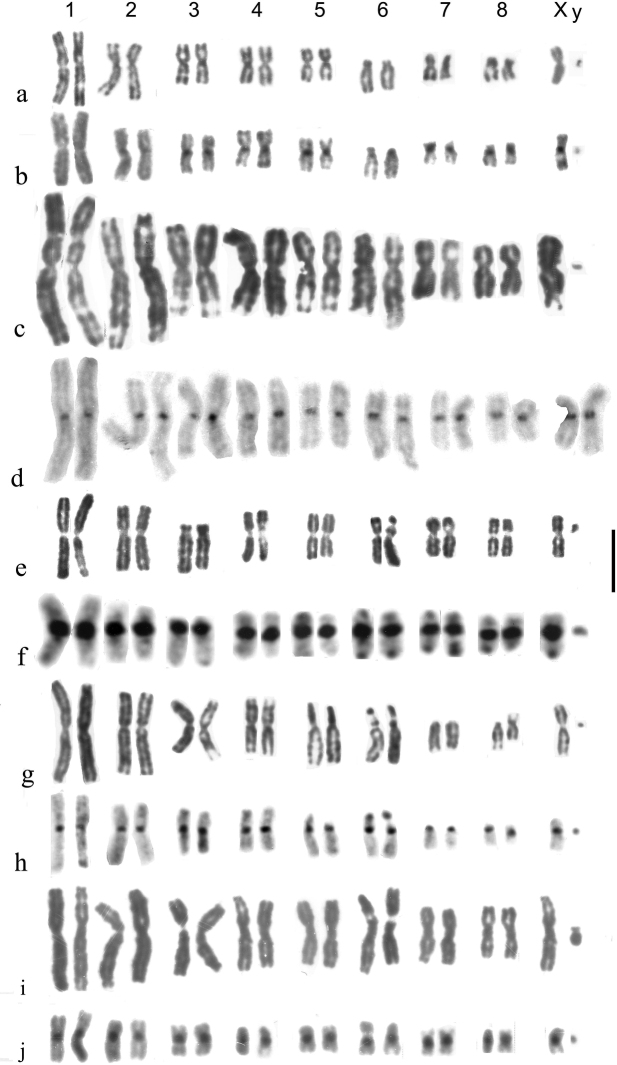
**a–j***Helophorus* str. Mitotic chromosomes arranged as karyotypes **a, b***H.liguricus*, ♂, Corfu, mid gut **a** Giemsa-stained **b** C-banded **c, d***H.maritimus*, embryos, France, Camargue **c** ♂, Giemsa-stained **d** ♀, C-banded **e, f***H.occidentalis*, mid gut, Spain, Province of Cáceres, Abadia **e** Giemsa-stained **f** C-banded **g, h***H.milleri*, ♂, mid gut, Corfu **g** Giemsa-stained **h** C-banded **i, j***H.syriacus*, ♂, mid gut, Israel **i** Giemsa-stained **j** C-banded. Scale bar: 15 µm.

**Figure 4.**
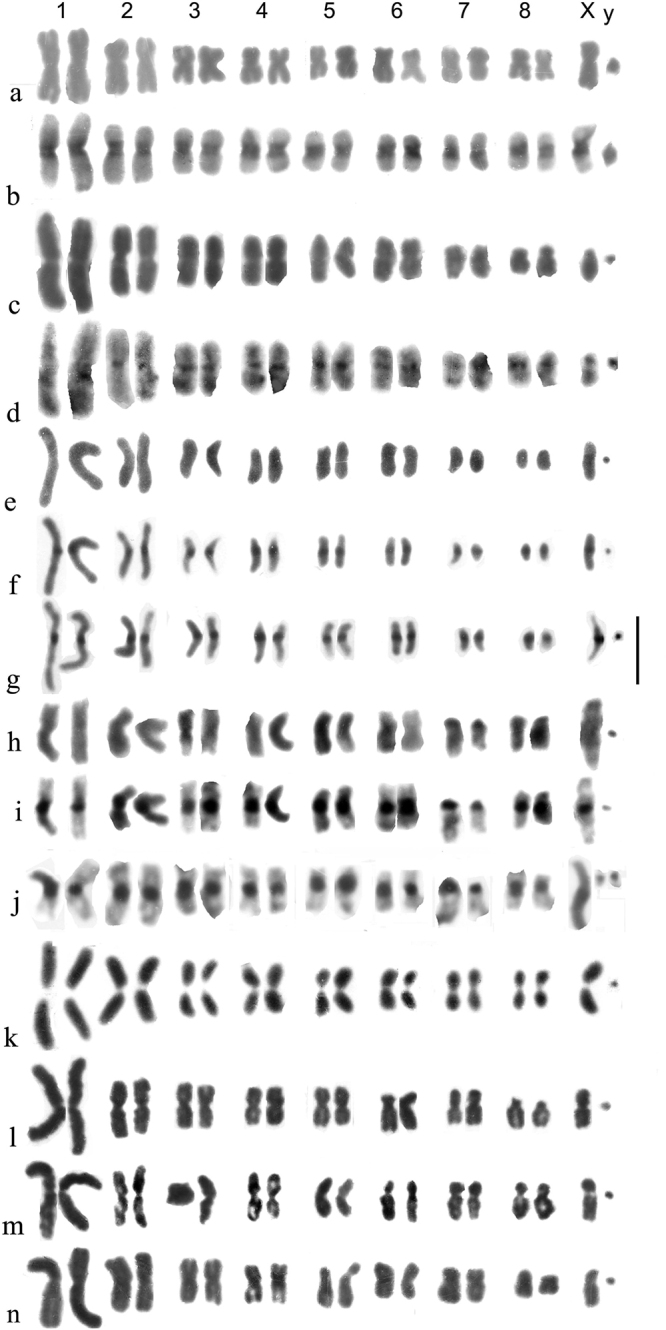
**a–n** Mitotic mid gut chromosomes of subgenera *Helophorus* s. str, *Gephelophorus* and *Eutrichelophorus*, arranged as karyotypes **a–h***Helophorus* s. str **a, b***H.oscillator*, ♂, Israel, Golan, Einot Summaga **a** Giemsa-stained **b** C-banded **c, d***H.hammondi*, China, Qinghai, Gangca **c** Giemsa-stained **d** C-banded **e–g***H.jaechi*, China, Sichuan, Xinduqiao **h–k***Gephelophorus***h–j***H.sibiricus*, ♂, China, Heilongjiang, Mishan **h** Giemsa-stained **i** the same nucleus C-banded **j** a different nucleus from the same specimen, C-banded **k***H.auriculatus*, ♂, Japan, Saitama prefecture near Tokyo, Giemsa-stained **l–n***Eutrichelophorus*, ♂, Giemsa-stained **l, m***H.micans***l** Crete, Rethymnon **m** Hungary **n***H.oxygonus*, Morocco, Ifrane. Scale bar: 15 µm.

**Figure 5.**
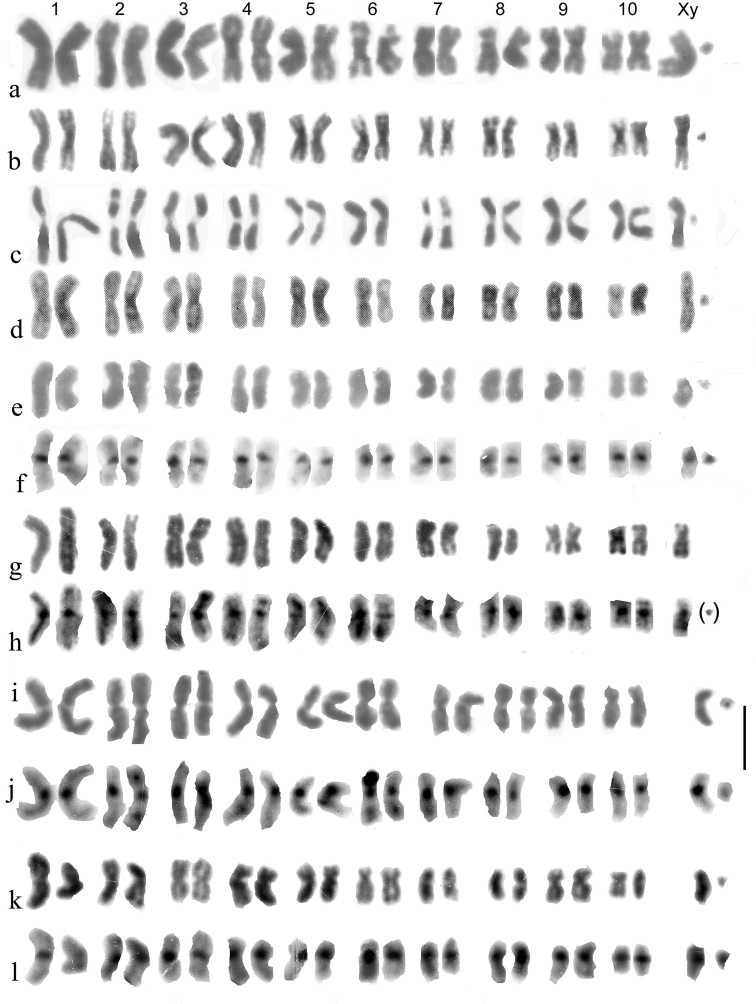
**a–l** Mitotic mid gut chromosomes of subgenera *Empleurus*, *Trichohelophorus* and *Lihelophorus*, arranged as karyotypes **a–c***Empleurus***a***H.nubilus*, ♂, Spain, Provincia de Salamanca, El Cubo, Giemsa-stained **b, c***H.rufipes*, ♂, Giemsa-stained **b** Spain, Provincia de Segovia, Santa Maria la Real de Nieva **c** England, Worcestershire **d***Trichohelophorusalternans*, ♂, Sardinia, Giemsa-stained **e–l***Lihelophorus*, ♂, China, Qinghai, Zuimatan **e, f***L.lamicola***e** Giemsa-stained **f** C-banded **g, h***L.ser***g** Giemsa-stained, the y chromosome lost from this preparation **h** C-banded, with the y from a different preparation **i–l***L.yangae***i, k** Giemsa-stained **i, j** and **k, l** the same nuclei, Giemsa-stained and C-banded. Scale bar: 15 µm.

**Figure 6.**
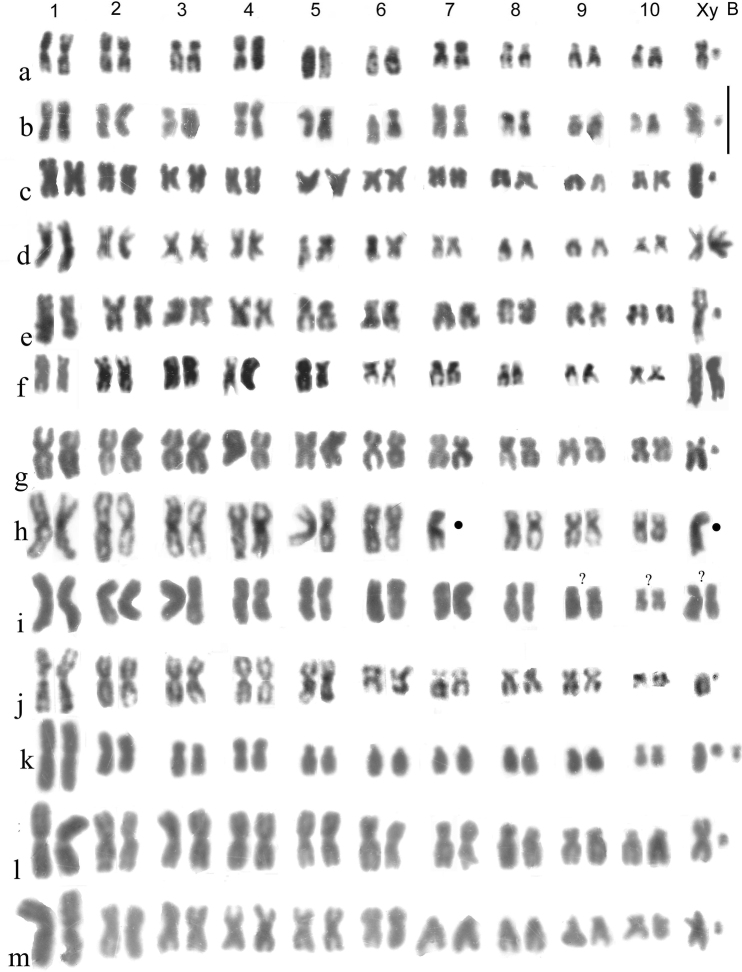
**a–m** Subgenus Rhopalohelophorus, informal grouping *Atractohelophorus*. Giemsa-stained mitotic mid gut chromosomes arranged as karyotypes **a, b***H.brevipalpis*, diploid ♂♂ **a** Spain, Province of León, Algadefe **b** Crete, Rethymnon **c, d***H.montenegrinus***c** Bulgaria, Rila **d** Italy, Stirone **e, f***H.glacialis***e** ♂, Spain, Provincia de Madrid, Peña Labra **f** ♀ Corsica, Haute-Corse, Restonica **g, h***H.leontis*, ♂, Spain, Province of Madrid, Peña Lara **i***H.dixoni*, ♀, Israel, Golan **j***H.biltoni*, Iran, Fars Province, Sishpir **k***H.nevadensis*, ♂, Spain, Province of Madrid, Peña Lara **l***H.korotyaevi*, ♂, Spain, Province of Cantabria, Puerto de Piedrasluengas **m***H.lewisi*, ♂, Israel, Golan, Einot Summaga. The positions of missing chromosomes are indicated by small black discs. Scale bar: 15 µm.

**Figure 7.**
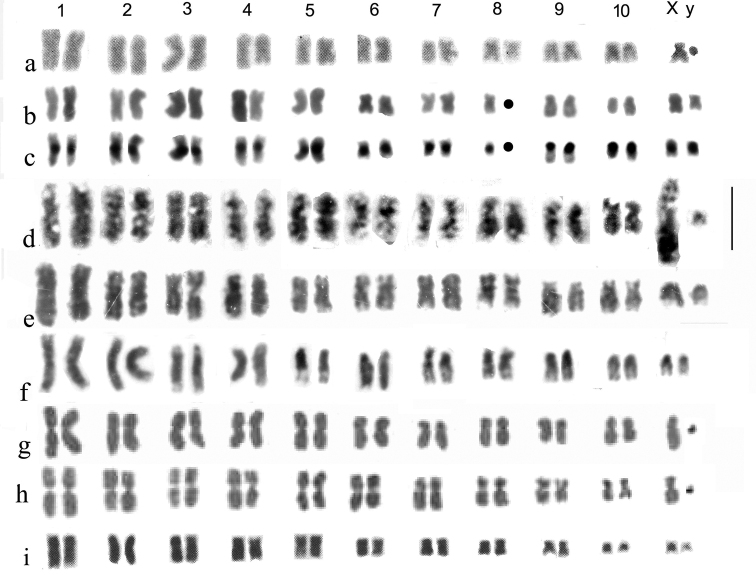
**a–i** Subgenus Rhopalohelophorus, species with 8-segmented antennae **a–c***H.nanus***a** ♂ embryo, France, Beaumont-sur-Sarthe, Giemsa-stained **b, c** ♀, mid gut, China, Heilongjiang, Mishan **b** Giemsa-stained **c** C-banded **d***H.redtenbacheri*, ♂, embryo, Russia, West Siberia, Karasuk, Giemsa-stained **e***H.pallidus*, ♀, embryo, Russia, West Siberia, Karasuk, Giemsa-stained **f***H.villosus*, ♀, Germany, Bavaria, Deggendorf, embryo, Giemsa-stained **g, h***H.pallidipennis*, ♂, embryo, Giemsa-stained **g** Cyprus **h** Crete **i***H.kervillei*, ♀, embryo, Giemsa-stained, Corfu. The positions of missing chromosomes are indicated by small black discs. Scale bar: 15 µm.

**Figure 8.**
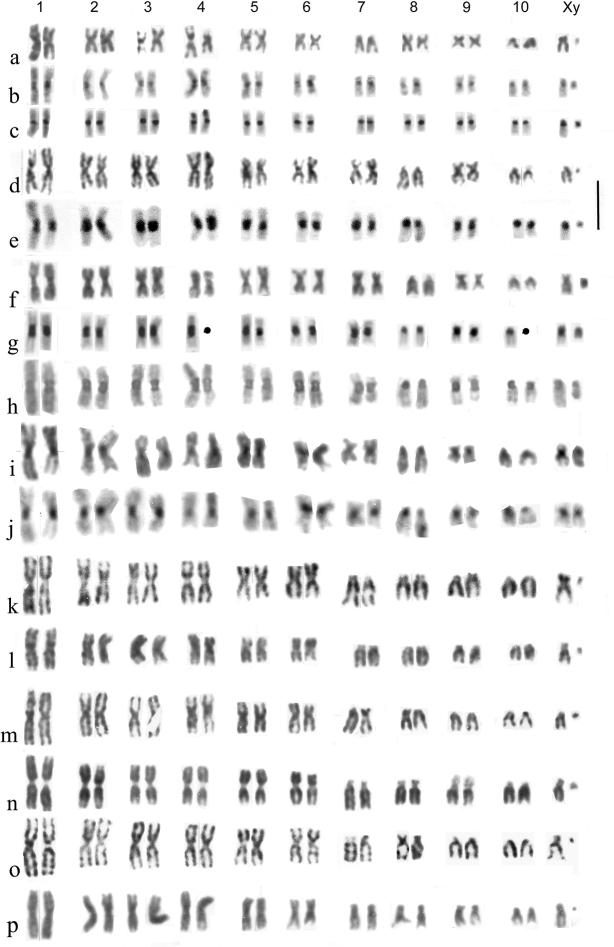
**a–p** Subgenus Rhopalohelophorus, *H.minutus*-group **a–c***H.minutus* ♂, embryos **a, b** England, Surrey, Runnymede **a** Giemsa-stained **b** C-banded **c** Spain Province of Segovia, Villacastín, C-banded **d, e***H.atlantis*, ♂, embryos, Morocco, Ifrane **d** Giemsa-stained **e** C-banded **f–j***H.calpensis*, Spain **f–h** ♂ Provincia de Cádiz, Tarifa, embryos **f** Giemsa-stained **g, h** C-banded **i, j** ♀, Province of Huelva, Coto Doñana, mid-gut **i** Giemsa-stained **j** the same nucleus, C-banded **k, l***H.paraminutus*, ♂, embryos, Giemsa stained **k** Russia, West Siberia, Karasuk **l** Austria, Neusiedler See area **m–p***H.lapponicus*, ♂ **m–o** embryos **p** mid gut **m** ♀, Spain, Province of Cantabria X ♂, Sweden, Västerbotten **n, o** Russia, West Siberia, Karasuk **n** treated with cycloheximide then Giemsa-stained **o** Giemsa-stained **p** Israel, Golan, Einot Summaga, Giemsa-stained. Scale bar: 15 µm.

**Figure 9.**
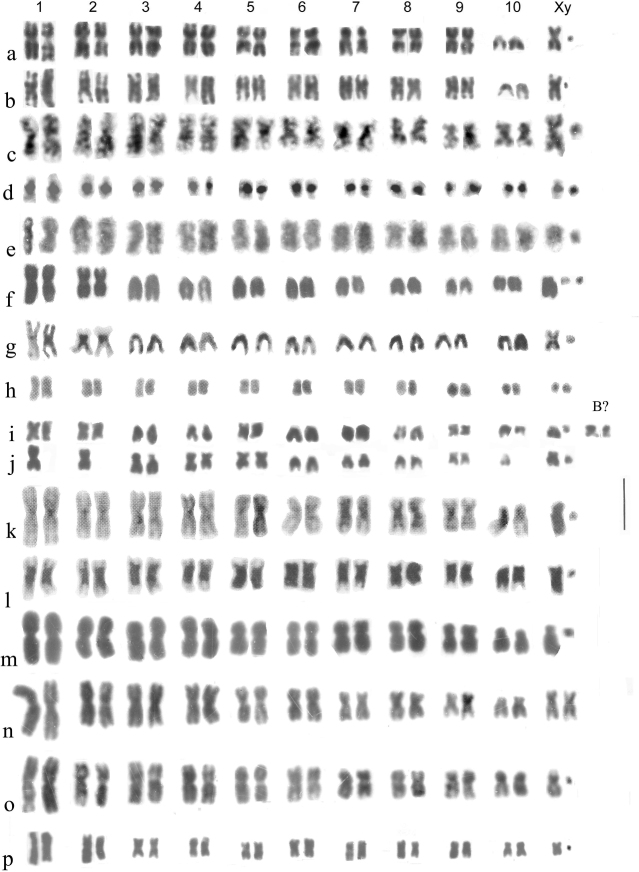
**a–p** Subgenus Rhopalohelophorus, various **a***H.fulgidicollis*, ♂, England, Hampshire, Lymington, embryo, trypsin-treated, Giemsa-stained **b***H.asturiensis*, ♂, France, Sarthe, Beaumont-sur-Sarthe, embryo, trypsin-treated, Giemsa-stained **c, d***H.kirgisicus*, ♂, Russia, West Siberia, Karasuk, embryos **c** Giemsa-stained but partially decomposed in polymerising resin **d** C-banded **e***H.similis*, ♂, Russia, West Siberia, Karasuk, embryo, Giemsa-stained but partially decomposed, phase-contrast **f***H.griseus*, ♂, Sweden, Öland, embryo, Giemsa-stained **g***H.granularis*, ♂, France, Sarthe, Beaumont-sur-Sarthe, embryo, Giemsa-stained **h***H.discrepans*, ♀, Spain, Pyrenees, embryo, Giemsa-stained **i, j***H.jocoteroi*, ♂, mid gut cells from the same paratype, Province of La Coruña, Esclavitud, Giemsa-stained **k***H.strigifrons*, ♂, France, Indre, Scoury, embryo, Giemsa-stained **l***H.asperatus*, ♂, France, Sarthe, Beaumont-sur-Sarthe, embryo, Giemsa-stained **m, n***H.pumilio*, Netherlands, Druten, embryos, Giemsa-stained **m** ♂ **n** ♀ **o***H.croaticus*, ♂, Netherlands, Druten, embryo, Giemsa-stained **p***H.cincticollis*, ♂, Morocco, Fes, embryo, Giemsa-stained. Scale bar: 15 µm.

**Figure 10.**
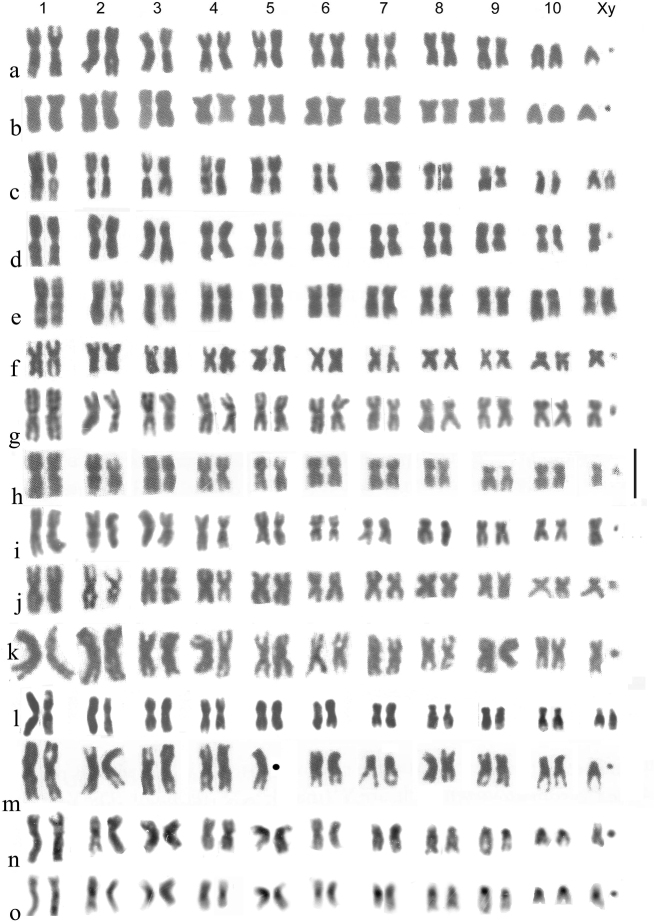
**a–o** Subgenus Rhopalohelophorus, mainly *H.flavipes* group **a–c***H.flavipes***a** ♂, England, Hampshire, New Forest, embryo, Geimsa-stained **b** ♂, Spain, Province of Madrid, Peña Lara, embryo, Giemsa-stained **c** ♀, Sweden, mid gut, Giemsa-stained **d–g***H.obscurus***d** ♂, Öland, embryo, Giemsa-stained **e** ♀, England, Surrey, Chobham Common, embryo, Giemsa-stained **f** ♂, France, Corsica, Ajaccio, mid gut, Giemsa-stained **g** ♂, Crete, Rethymnon, embryo, Giemsa-stained **h, i***H.algiricus* ♂, Morocco, Ifrane, mid gut, Giemsa-stained **j, k***H.subarcuatus* ♂, Italy, Sardinia, Mandas, mid gut, Giemsa-stained **l, m***H.seidlitzi*, mid gut, Spain **l** ♀, Province of Segovia, Cuéllar, embryo, Giemsa-stained **m** ♂, Province of León, Algadefe, mid gut, Giemsa-stained **n, o***H.browni* ♂, China, Heilongjiang, Qitaihe, mid gut **n** Giemsa-stained **o** the same nucleus C-banded. The position of missing chromosomes is indicated by a small black disc. Scale bar: 15 µm.

**Figure 11.**
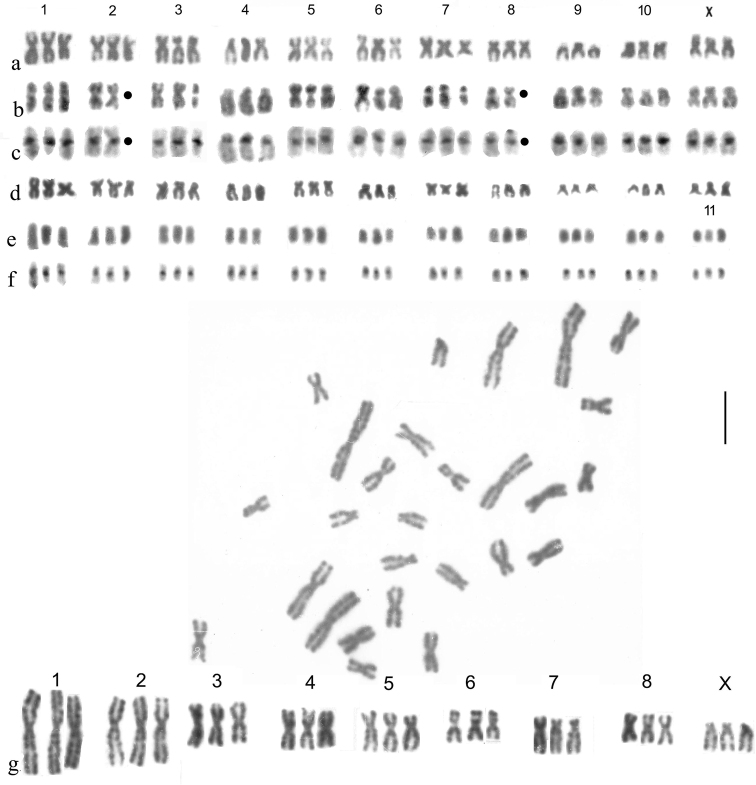
**a–g** Triploid females, Giemsa-stained **a–d***H.brevipalpis***a** Spain, Province of León, Algadefe **b, c** Italy, Sologno **b** Giemsa-stained **c** the same nucleus C-banded **d** Italy, Ponte Scipione **e, f***H.orientalis*, China, Heilongjiang, Qitahe **e** Giemsa-stained **f** the same nucleus C-banded. No male *H.orientalis* was available so the X chromosome cannot be identified **g***H.aequalis*, a solitary triploid embryo found among numerous normal diploids from egg cocoons from France, Cantal, St Flour. The positions of missing chromosomes are indicated by small black discs. Scale bar: 15 µm.

**Figure 12.**
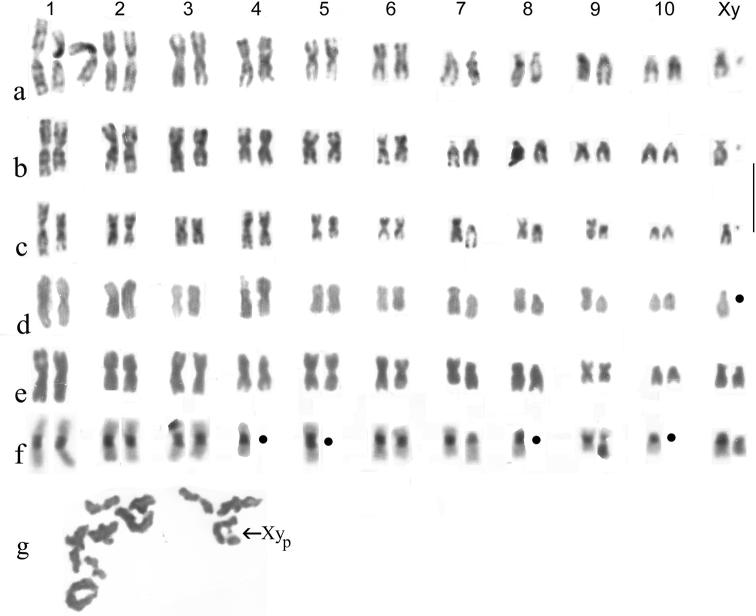
**a–f** experimental Hybrids **a, b** ♂ hybrid embryos, *H.lapponicus* ♀ lab-reared from Karasuk X ♂ *H.paraminutus*, from Karasuk, with the shorter replicate of autosome 1 shown in its natural position (on the right) and “straightened” (centre) **c, d** ♂ hybrid embryos, *H.minutus* ♀, lab-reared from Egham, Surrey X ♂, *H.paraminutus*, wild-caught, Austria **e, f** hybrid embryos **e** ♂ **f** ♀, *H.minutus* ♀, lab-reared from Egham, Surrey X ♂, *H.calpensis*, wild-caught, Tarifa, Spain. The suggested positions of missing chromosomes are indicated by small black discs **g** Meiosis, first metaphase from a ♀ *H.lapponicus* X ♂*H.paraminutus* hybrid, showing 10 bivalents + Xy_p_ sex chromosomes (labelled). Scale bar: 15 µm.

Work on *H.aquaticus* and *H.aequalis* required chromosomally verified material to establish the extent of their morphological variation, especially of the aedeagus. To begin with, testes of freshly emerged adults were used as a source of mitotic chromosomes, and this solved the problem. Later the technique was extended to mid gut, where undifferentiated cells in the mid gut crypts undergo mitosis to replace epithelial cells lost in the course cells of food-digestion. I had been steadily accumulating karyotypes of various *Helophorus* species, and 1989 I produced a preliminary Atlas, covering 31 species, for the Balfour-Browne Club Newsletter (Angus 1989). This work has continued, often focusing on groups of similar-looking species requiring taxonomic clarification. So now there is a new version of the Atlas with 62 species. This is presented here, with Figs 2–12.

## ﻿Atlas of *Helophorus* chromosomes

*Helophorus* species divide into two karyotype-groups, those with eight pairs of autosomes plus Xy_p_ sex chromosomes (the so-called “parachute-association” with the very small y chromosome attached to the X by a nucleolus or cytoplasmic vesicle, described by John & Lewis (1960) and with the possibility that the cytoplasmic vesicle was not always a true nucleolus (Juan et al. 1993) (subgenera *Helophorus* s. str., *Gephelophorus* Sharp, 1915 and *Eutrichelophorus* Sharp, 1915), and those with 10 pairs of autosomes plus Xy_p_ sex chromosomes (subgenera *Empleurus* Hope, 1838, *Trichohelophorus* Kuwert, 1886, *Lihelophorus* Zaitzev, 1908 and *Rhopalohelophorus* Kuwert, 1886).

**Subgenus Helophorus s. str.**

Figs 2a–j, 3a–j, 4a–g

Species of *Helophorus* s. str. divide morphologically into three groups, the *H.aquaticus* group with the last fixed abdominal segment bearing small but clearly square-ended teeth, the *H.grandis* Illiger, 1798 group, with much larger teeth and the *H.bergrothi* J. Sahlberg, 1880 group, in which the abdominal sternite is crinkled apically but with the shape of the teeth not really discernible except sometimes in cleared, slide-mounted preparations (Angus 1970a). One particularly distinctive feature of the karyotype, originally discovered in *H.aequalis*, is the presence of a distinct secondary constriction, confirmed by silver staining as the site of a NOR (Angus 1982). In *H.aequalis* this chromosome goes as pair 6 in the row of chromosomes in the karyotype, and in other species the NOR-bearing chromosome is placed as pair 6 for ease of comparison.

*H.aquaticus* (Fig. 2a, b) . The NOR-bearing chromosome 6 is about as long as pair 3, depending on the degree of opening of the secondary constriction. The centromeric C-bands are small (see Fig. 1) and the X chromosome is submetacentric.

*H.thauma* Angus et Toledo, 2010 (Fig. 2c, d) . An Italian species very closely resembling *H.aequalis* but distinguished chromosomally by the NOR-bearing chromosome 6 being as long or longer than autosome 3.

*H.aequalis* (Fig. 2e, f) . The centromeric C-bands are fairly strong, and autosome 7 and the X chromosome are subacrocentric. The NOR-bearing chromosome 6 is shorter than 5, but about the same length as pair 7. Other details of comparison with *H.aquaticus* are given in the section discussing banding, and by Angus (1982).

*H.grandis* (Fig. 2g–j) . Although this is the first of the big-toothed group of species, it has been found by Martin Fikáček (*pers*. *comm*.16.VII.2023) in the course of his ongoing DNA analysis, to be the sister-species of *H.aequalis*, and chromosomally this is supported by the size and shape of the NOR-bearing autosome 6. The centromeric C-bands are clearly larger than in *H.aequalis*, and there may be an interstitial C-band in the middle of the long arm of the acrocentric X chromosome, which is thus polymorphic for long and short forms (Fig. 2h–j). Silver-staining (Fig. 2i) shows the interstitial C-band behaving rather differently from the centromeric one. The short form matches the *H.aequalis* X. Autosome 5 is polymorphic for a pericentric inversion, and may be either metacentric as in *H.aequalis*, or acrocentric. Smith (1960) correctly recorded Canadian “*H.aquaticus*” (actually *H.grandis*, a Palaearctic species introduced in Canada) as having 18 chromosomes including Xy_p_. He also gave this number for *H.oblongus* LeConte, 1850, a Holarctic species of the subgenus Rhopalohelophorus, the group with 8-segmented antennae, and therefore expected to have 22 chromosomes including Xy_p_. This result needs to be checked.

*H.liguricus* Angus, 1970 (Fig. 3a, b) . The position of the NOR is not clear but it may be at the distal end of the short arm of autosome 7. Autosome 6 is acrocentric and the X chromosome is a smallish metacentric, similar in size to autosomes 4 and 5. The C-bands vary in size between the chromosomes, apparently absent from pair 1, very small in pairs 2 and 3, slightly larger in the others.

*H.maritimus* Rey, 1885 (Fig. 3c, d) . The centromeric C-bands are small, the NOR may be located at the distal end of the long arm of autosome 6, and the X chromosome is a fairly long metacentric, about as long as autosomes 4 and 5.

*H.occidentalis* Angus, 1983 (Fig. 3e, f). Known from the southern parts of Spain and Portugal, and from Morocco. NOR-bearing autosome 6 is small, about as long as pairs 3–5, slightly longer than pairs 7 and 8. The metacentric X chromosome is slightly larger than autosomes 7 and 8, but smaller than 6. The centromeric C-bands are particularly heavy in all chromosomes except the dot-like y, pairs 6 and 7 have terminal C-bands at both ends and pairs 1 and 8 are polymorphic for the presence of a small C-band at the end of their long arms.

*H.milleri* Kuwert, 1886 (Fig. 3g, h). Characterised by small centromeric C-bands, autosome 5 being acrocentric, autosome 6 with its NOR located medially on the short arm and matching its position, being about the same size as autosome 5 but longer than 7. The distal part of the short arm, beyond the NOR, is heterochromatic, and there may be a C-band at the distal end of the long arm. Autosome 8 is polymorphic for a pericentric inversion, resulting in metacentric and acrocentric forms. The X chromosome is metacentric, longer than autosomes 7 and 8, but slightly shorter than 6. Described from Corfu, this species is widespread in the central Mediterranean area.

*H.syriacus* Kuwert, 1885 (Fig. 3i, j). The NOR-bearing autosome 6 is as long as pair 3 and the metacentric X chromosome is also long, as pair 4. The centromeric C-bands are fairly heavy, smaller and fainter on pairs 2 and 8. My material is from Israel, but this species is widely distributed from western Anatolia (and adjacent Greek islands) east to the mountains of Kazakhstan (Aksu-Dzhabagli).

*H.oscillator* Sharp, 1915 (Fig. 4a, b). Originally placed in *Trichohelophorus* Kuwert, 1886 by Sharp, this species was transferred to *Helophorus* s. str. by Angus et al. (2019), largely because of its karyotype. The chromosomes are all metacentric, with fairly large centromeric C-bands and the small y chromosome is also heavily C-banded. The intensity of the bands varies but this may be an experimental artefact.

*H.hammondi* Angus, 1970 (Fig. 4c, d). Autosomes 1–7 are more or less metacentric with moderate centromeric C-bands. The C-banding of pair 4, with a weak band in the middle of the short arm, suggests this may be the site of the NOR. Autosome pair 8 and the X chromosome are subacrocentric, with the X chromosome about the same size as pair 7.

*H.jaechi* Angus, 1995 (Fig. 4e–g). The general layout of the chromosomes is similar to that of *H.hammondi*, but autosome pair 4 is subacrocentric and the X chromosome is distinctly larger, about as long as pair 3. The centromeric C-bands are small but distinct.

**Subgenus Gephelophorus**

Fig. 4h–k

*H.sibiricus* Motschulsky, 1860 (Fig. 4h–j). Autosomes 1–6 and 8 are metacentric, 7 is acrocentric and the metacentric X chromosome is the longest in the nucleus. All the chromosomes, except the tiny y have heavy centromeric C-bands, and pair 7 has a size polymorphism with, in Fig. 4i, the longer replicate of pair 7 with an apparent C-band in the middle of its long arm. The somewhat fainter appearance of this band matches that of *H.grandis* when silver-stained (Fig. 2i). The nucleus shown in Fig. 4j has 2 y chromosomes.

*H.auriculatus* Sharp, 1884 (Fig. 4. k). All the autosomes, and the X chromosome, are metacentric, with the X chromosome about as long as pair 3. The y is very small, dot-like. No C-banding is available, but the distinct centromeric gaps suggest the presence of large C-bands.

**Subgenus Eutrichelophorus**

Fig. 4l–n

*H.micans* Faldermann, 1835 (Fig. 4l, m). Ongoing DNA investigation by Martin Fikáček (*pers. comm*.16.VII.2023) associates this species with *Helophorus* s. str., in agreement with its chromosome number. Autosome pairs 1–5 are metacentric, 6, 7 and the X chromosome are borderline submetacentric/subacrocentric, and pair 8 is subacrocentric. The y chromosome is very small, dot-like.

*H.oxygonus* Bedel, 1881 (Fig. 4n). A very similar karyotype to that of *H.micans* but with pair 5 borderline acrocentric/subacrocentric and possibly longer than pair 4, and pair 6 metacentric.

### ﻿Subgenera with karyotypes of 20 +Xy_p_

**Subgenus Empleurus**

Fig. 5a–c

*H.nubilus* Fabricius, 1777 (Fig. 5a). All the autosomes, and the X chromosome are metacentric, with the X chromosome about as long autosome 2. The y chromosome is a dot. No C-banding is available, but this Giemsa-stained karyotype suggests that at least some of the autosomes have large centromeric C-bands.

*H.rufipes* Bosc, 1791 (Fig. 5b, c). The general layout of the karyotype is similar to that of *H.nubilus*. This is especially clear in the Spanish specimen (Fig. 5b).

**Subgenus Trichohelophorus**

*H.alternans* Gené, 1836 (Fig. 5d). All the autosomes and the X chromosome are metacentric, with pairs 9 and 10 approaching the border with submetacentric. The y is a dot.

**Subgenus Lihelophorus**

Fig. 5e–l

The three species of this subgenus are endemic to the Tibetan Plateau. They are unique in *Helophorus* in having the outermost elytral interval (interval 10) completely flat, so that there is no trace of pseudepipleura outside the elytral epipleurs. The combination of elytral intercalary (scutellary) striae and asymmetrical apical segments of the maxillary palpi suggests association of *Lihelophorus* with *Helophorus* s. str. but the chromosomes show that this is not the case. The subgenus was reviewed by Angus et al. (2016).

*H.lamicola* Zaitzev, 1908 (Fig. 5e, f). All the autosomes are more or less metacentric with distinct centromeric C-bands. The X chromosome, similar in size to autosome 10, is subacrocentric, again with a distinct centromeric C-band. The small, almost dot-like y chromosome also has a small C-band.

*H.ser* Zaitzev, 1908 (Fig. 5g, h). The general layout of the karyotype is very similar to that of *L.lamicola*. The X chromosome is slightly larger and with slightly longer short arms. The y chromosome is dot-like,

*H.yangae*Angus et al., 2016 (Fig. 5i–l). Autosomes 4–6 are clearly less metacentric than in the other two species, and the X chromosome is slightly larger, similar in size to autosome pair 7 rather than pair 8.

**Subgenus Rhopalohelophorus**

Figs 6a–m, 7a–i, 8a–p, 9a–p, 10a–o, 11a–g

Informal group *Atractohelophorus* (Fig. 6a–m). *Atractohelophorus* refers to the small species with symmetrically oval apical segments on their maxillary palpi. In most of Europe by far the commonest species is *H.brevipalpis* Bedel, 1881, and many of the other species tend to be associated with mountains.

*H.brevipalpis*, bisexual, diploid (Fig. 6a, b). Autosome pairs 1, 2, 4 and 7, and the X chromosome are metacentric, 3, 6, 8, 9 and 10 are borderline acrocentric/subacrocentric and 6 is acrocentric in the Spanish specimen (Fig. 6a) polymorphic for a pericentric inversion, either subacrocentric or metacentric in the Cretan one (Fig. 6b). The y chromosome is dot-like. For parthenogenetic triploids see Fig. 11a–d.

*H.montenegrinus* Kuwert, 1885 (Fig. 6c, d). The karyotype is very like that of *H.brevipalpis*, but autosome pair 3 is metacentric and 6 is submetacentric.

*H.glacialis* Villa et Villa, 1833 (Fig. 6e, f). Autosome pairs 1–4, 6 and 10 are metacentric and 5 and 7–9 are subacrocentric. The metacentric X chromosome is clearly the longest in the nucleus, a feature shared with *H.redtenbacheri* Kuwert, 1885 (Fig. 7d). The y chromosome is small, almost dot-like.

*H.leontis* Angus, 1985 (Fig. 6g, h). Autosomes 1–7 and 10 are metacentric, 8 and 9 and the X chromosome are submetacentric. The X chromosome is about the same size as autosomes 6 and 7.

*H.dixoni* Angus, 1987 (Fig. 6i). No male karyotype is available, so the X chromosome cannot be identified. Chromosomes 1–8, on the arrangement adopted here, match those of *H.leontis*. Of the smaller autosomes, one, placed as pair 10, is clearly smaller than anything in the *H.leontis* karyotype. In the current arrangement the X chromosome would be smaller than that of *H.leontis*, about the same size as pair 8.

*H.biltoni*Angus et al., 2005(Fig. 6j). Autosomes 1–6 match those of *H.leontis* and *H.dixoni*, but pairs 7–9 are smaller, and the small autosome 10 matches chromosome 10 of *H.dixoni*. The X chromosome is a small acrocentric, clearly smaller than the *H.leontis* X chromosome and not matching any of the *H.dixoni* chromosomes.

*H.leontis*, *H.dixoni* and *H.biltoni* are a group of species which cannot be separated by their aedeagal morphology, though their body-forms differ. Their karyotypes leave no doubt that they are separate species.

*H.nevadensis* Sharp, 1916 (Fig. 6k). Autosomes 1, 2 and 4 are metacentric, with pair 1 about twice the length of pair 2. The remaining autosomes, and the X chromosome, are acrocentric to subacrocentric. The X chromosome is about the same length as autosome 8. One B-chromosome is present, about the same size as the diminutive y. I have seen this chromosome in both the males from which I have obtained karyotypes. I have no female preparations and cannot say which of the tiny chromosomes is the y and which is a B. They are both about a third of the length of the X.

*H.korotyaevi* Angus, 1985 (Fig. 6l). Autosomes 1–5, and the X chromosome are metacentric and autosomes 6–10 are submetacentric to subacrocentric. The X chromosome is about the same size as autosomes 6 and 7. The diminutive y chromosome is about a third of the length of the X.

*H.lewisi* Angus, 1985 (Fig. 6m). Autosomes 1–6 and the X chromosome are metacentric, 7 and 8 are acrocentric and 9 and 10 are subacrocentric. The X chromosome is about as long as autosome 6 and the diminutive y is about a quarter that length.

#### *Rhopalohelophorus*, species with 8-segmented antennae

Fig. 7a–i

Not a natural group, but convenient.

*H.nanus* Sturm, 1836 (Fig. 7a–c). Probably the most widely distributed species in the Palaearctic, from Britain, Ireland and France in the west to the Russian Far East (Primorye) in the east. Autosomes 1–8 are metacentric, 9 and 10, along with the X chromosome are subacrocentric. The diminutive, almost dot-like, y chromosome is about a third of the length of the X, itself one of the shortest in the nucleus. There appears to be no morphological difference between the French and Chinese specimens figured here.

*H.redtenbacheri* (Fig. 7d). This is one of the preparations made at Karasuk in 1982 and slide-mounted using polymerising UV setting resin. Unfortunately, it had deteriorated badly before it was photographed. Nevertheless, the main morphological features of the chromosomes can be discerned. As mentioned in the discussion of *H.glacialis* (Fig. 6f, g), this is a species whose X chromosome is clearly the longest in the nucleus. Autosomes 1–3, 5–7, and the X chromosome, are metacentric. Autosomes 4 and 8–10 are submetacentric.

*H.pallidus* Gebler, 1830 (Fig. 7e) As no male karyotype is available the X chromosome cannot be recognised. Chromosomes 1 and 2 and 5–8 are metacentric, 3 and 4 are submetacentric, and the rest are subacrocentric.

*H.villosus* Duftschmid, 1805 (Fig. 7f). No male karyotype is available so the X chromosome cannot be identified. Chromosomes 1–4 are metacentric, 5, 7 and 9 are submetacentric, 8 is borderline submetacentric/subacrocentric and 6, 10 and 11 are acrocentric to subacrocentric.

*H.pallidipennis* Mulsant et Wachanru, 1852(Fig. 7g, h). Autosome pairs 1–6 are metacentric, while 7- 10 and the X chromosome are subacrocentric. The X chromosome is about the same length as autosomes 7 and 8 and the y is dot-like.

*H.kervillei* d’Orchymont, 1932 (Fig. 7i). No male karyotype is available so the X chromosome cannot be recognised. Chromosomes 1–8 are metacentric, 9 is submetacentric and 10 and 11 are apparently metacentric, but very small. This species, like *H.kirgisicus* Kniž, 1914, has only two larval instars. Angus (1992a) regarded this as a form of *H.pallidipennis*, which he therefore described as having only two larval instars. Only when information on Cretan and Cypriot *H.pallidipennis* revealed not only a different karyotype but also a third larval instar, did the truth become apparent (Angus, 1998).

#### *Rhopalohelophorus*, the *H.minutus* Fabricius, 1775 group

Fig. 8a–p

For experimental hybrids see later, Fig. 12.

*H.minutus* (Fig. 8a–c). Autosomes 1–7 and 9 are metacentric, pairs 8 and 10, and the X chromosome are subacrocentric. The X chromosome is about the same size as pair 8 and the diminutive y is almost dot-like, perhaps metacentric. One autosome has a modified apical part of the short arm, probably the site of a NOR, very pale in one replicate of the Giemsa-stained pair, slightly C-banded in the English C-banded preparation (Fig. 8b) and more strongly so in the Spanish one (Fig. 8c). This autosome was originally placed as pair 2 (Angus, 1986) but, bearing in mind the large variation in the degree of condensation of this autosome and a more averaged interpretation of its length, placing it a pair 4 seems more appropriate. This also agrees with its position in the related *H.atlantis* and *H.calpensis*. Autosome 9, an even metacentric, often takes the form of a multiplication sign (X) and is one of the landmarks of the *H.minutus* karyotype. Angus (1986) reversed the positions of pairs 8 and 9, to place the metacentrics before the submetacentrics. This is in fact unhelpful and counter to the Relative Chromosome Length data presented in Table 1 of Angus (1986). The positions of autosomes 7 and 8 have also been reversed in the light of study of more material, including hybrids. *H.minutus* is widely distributed over much of Europe.

**Table 1.** Summary of the data.

Subgenus/species	Karyotype/peculiarities	Reference
*Helophorus* s. str.	2n = 16 + Xy_p_	
*H.aquaticus*		Angus 1982
*H.thauma*		Angus and Toledo 2010
*H.aequalis*		Angus 1982
*H.grandis*	Autosome 5 metacentric or acrocentric, polymorphic for a pericentric inversion. X chromosome with a length polymorphism associated with an interstitial C-band. 1 or 2 B-chromosomes	Angus 1983
*H.liguricus*		Angus 1989
*H.maritimus*		Angus 1983
*H.occidentalis*		Angus 1983
*H.milleri*	Autosome 8 metacentric or acrocentric, polymorphic for a pericentric inversion.	Angus 1989
*H.syriacus*		Angus 1989
*H.oscillator*		Angus 1989
*H.hammondi*		Angus 2015
*H.jaechi*		This paper
H. (Gephelophorus)	2n = 16 + Xy	
*H.auriculatus*		Angus 2015
*H.sibiricus*	Autosome 7 with a length polymorphism associated with interstitial heterochromatin	Angus 2019; This paper
H. (Eutrichelophorus)	2n = 16 + Xy	
*H.micans*		Angus 2015
*H.oxygonus*		Angus 2015
H. (Empleurus)	2n = 20 + Xy	
*H.nubilus*		Angus 2015
*H.rufipes*		Angus 2015
H. (Trichohelophorus)	2n = 20 + Xy	
*H.alternans*		Angus 1989
H. (Lihelophorus)	2n = 20 + Xy	
*H.lamicola*		Angus et al. 2916
*H.ser*		Angus et al. 2016
*H.yangae*		Angus et al. 2016
H. (Rhopalohelophorus)	2n = 20 + Xy	
*H.brevipalpis*	Diploid: Autosome 5 metacentric or acrocentric, polymorphic for a pericentric inversion. Triploid ♀♀: 3n = 30 + 3X	Angus 1992b
*H.montenegrinus*		This paper
*H.glacialis*		This paper
*H.leontis*		Angus et al. 2005
*H.dixoni*		Angus et al. 2005
*H.biltoni*		Angus et al. 2005
*H.nevadensis*	B-chromosomes	This paper
*H.korotyaevi*		This paper
*H.lewisi*		This paper
*H.nanus*		Angus 1989, 2015
*H.redtenbacheri*		Angus 1989
*H.pallidus*		Angus 1989
*H.villosus*		Angus 1989
*H.pallidipennis*		Angus 1998
*H.kervillei*		Angus 1998
*H.minutus*		Angus 1986
*H.atlantis*		Angus and Aouad 2009
*H.calpensis*		Angus 1988
*H.paraminutus*		Angus 1986
*H.lapponicus*		Angus 1986
*H.fulgidicollis*		Angus 1989
*H.asturiensis*		Angus 1989
*H.kirgisicus*		Angus 1989
*H.similis*		Angus 1989
*H.griseus*		Angus 1989
*H.granularis*		Angus 1989
*H.jocoteroi*	2 B-chromosomes	Angus and Diaz Pazos 1990
*H.strigifrons*		Angus 1989
*H.asperatus*		Angus 1989
*H.pumilio*		This paper
*H.croaticus*		This paper
*H.cincticollis*		This paper
*H.flavipes*		Angus 1989, 1996
*H.obscurus*		Angus 1989, 1996
*H.algiricus*		Angus 1996
*H.subarcuatus*		Angus 1996
*H.seidlitzi*		Angus 1989, 1996
*H.browni*		Angus 2019
*H.orientalis*		Angus and Jia 2020
**Hybrids**		
♀*H.lapponicus* X ♂*H.paraminutus*	2n = 20 + Xy	Angus 1986
♀*H.minutus* X ♂*H.paraminutus*	2n = 20 + Xy	Angus 1986
♀*H.minutus* X ♂ *H.calpensis*	2n = 20 + Xy	Angus 1988

*H.atlantis* Angus et Aouad, 2009 (Fig. 8d, e). The karyotype is very similar to that of *H.minutus*, but the NOR appears to be at the distal end of the short arm of pair 4, pair 9 is less evenly metacentric and the X chromosome is as small as pair 10. The centromeric C-bands are noticeably heavy. This species is known from the Moyen Atlas of Morocco.

*H.calpensis* Angus, 1988 (Fig. 8f–j). The karyotype is very similar to that of *H.atlantis*, the most obvious difference being the size of the y chromosome, acrocentric and about half the length of the X. The position of the NOR-bearing chromosome is not easy to establish due to irregularities in condensation, but it appears to belong in position 4, as in *H.minutus* and *H.atlantis*. *H.calpensis* is so far known only from southernmost Spain, Tarifa and the Coto Doñana.

*H.paraminutus* Angus, 1986 (Fig. 8k, l). This species was initially recognised by Angus (1986) because it has a karyotype was apparently indistinguishable from that of *H.lapponicus* Thomson, 1858 but an egg cocoon like that of *H.minutus*, not *H.lapponicus*. Also, the beetles looked more like *H.minutus* than *H.lapponicus*, though they were often larger. Fig. 8k, l shows chromosomes from trypsin-treated Giemsa-stained embryos. Autosome pairs 1–6 are metacentric, while the others, and the X chromosome, are acrocentric to subacrocentric. The X chromosome is about the same size as autosome 7 and the y chromosome is dot-like. None of the metacentric chromosomes shows any indication of a terminal NOR.

*H.lapponicus* (Fig. 8m–p). The karyotypes shown in m and o are from embryos, m from a Spanish female crossed with a Swedish male, and o from Karasuk. The arrangement and banding patterns of the chromosomes appear identical. Note that one replicate of autosome in m has been damaged in the course of preparation. As mentioned above, the arrangement appears to be the same as that of *H.paraminutus*. Fig. 8n shows a preparation from a Karasuk embryo which was treated *in vitro* with cycloheximide. There is no trace of banding but autosome 9 shows some extension of the short arm, suggesting that this may be the site of the NOR. Fig. 8p shows a karyotype from a mid-gut cell of an Israeli specimen. The sequence of sizes and shapes of the chromosomes appears the same as in the other material.

#### *Rhopalohelophorus*, various species

*H.fulgidicollis* Motschulsky, 1860 (Fig. 9a). A trypsin-treated Giemsa-stained preparation from an embryo. No banding has resulted. Autosomes 1–5 and 8, 9 and the X chromosome are metacentric. The X chromosome is slightly shorter than pair 5, and the y is dot-like. Pairs 6 and 7 are submetacentric and the rounded condensed appearance of the short arm of 7 suggests this may be the site of the NOR. Pair 10 is a short acrocentric.

*H.asturiensis* Kuwert, 1885 (Fig. 9b). The karyotype appears very similar to that of *H.fulgidicollis*, though the beetles and their aedeagi are quite distinctly different.

*H.kirgisicus* Kniž, 1914 (Fig. 9c, d). This is another of the preparations which had partially decomposed in the polymerising resin. Autosomes 1–3, 5, 6 8, 10 and the X chromosome are metacentric and 4, 7 and 9 are submetacentric. The X chromosome is almost as large as autosome 1 and the y is very small, about a sixth the length of the X. One replicate of autosome 2 has the shorter arm expanded and the short arms of autosome 8 look as though as though they have small secondary constrictions. The C-banded karyotype (Fig. 9e) shows moderate centromeric C-bands on the larger chromosomes (1–5) and the X chromosome, but the chromosomes are too condensed for the banding of the smaller ones to be established.

*H.similis* Kuwert, 1887 (Fig. 9e). Another decomposed preparation, this time viewed under phase contrast. The karyotype seems very like that of *H.kirgisicus*, but with a shorter X chromosome.

*H.griseus* Herbst, 1793 (Fig. 9f). A very distinctive karyotype with autosomes 1 and 2 metacentric and all the others, as well as the X chromosome, acrocentric, with the X about as long as autosome 6. The y chromosome, almost dot-like, is about a third of the length of the X, and there is a similarly small B-chromosome in this individual.

*H.granularis* (Linnaeus, 1760) (Fig. 9g). As published by Angus (1989) the X chromosome was one of the longer acrocentrics (as in *H.griseus*, Fig. 9f) and autosome 8 was regarded as polymorphic for a pericentric inversion. Here a different arrangement, suggested by an anonymous referee, is adopted. This places the single metacentric as the X chromosome and autosomes 3–10 as acrocentrics, as in *H.griseus*. This should be checked using fresh material, especially females, but it is adopted here, not least because it makes fewer assumptions.

*H.discrepans* Rey, 1885 (Fig. 9h). No male karyotype is available, so the X chromosome cannot be identified. Chromosomes 1–8 are more or less metacentric, 9–11 acrocentric to subacrocentric.

*H.jocoteroi* Angus et Diaz Pazos, 1991 (Fig. 9i, j). Mid gut preparations from a single male. A karyotype of 12 pairs of chromosomes, including two presumed B-chromosomes. The karyotype shown in Fig. 9j appears to be complete, while that in k is incomplete but shows the shapes of some of the chromosomes more clearly. Autosomes 1, 2 and 9 are metacentric, 4 and 5 are submetacentric and the others, as well as the X chromosome, are acrocentric to subacrocentric. The X chromosome is about the same size as autosome 7 and the y is a dot. The smallest chromosomes, presumed to be Bs are about the size of the X chromosome, though less substantial, and appear to be acrocentric.

*H.strigifrons* Thomson, 1868 (Fig. 9k). Autosomes 1–7 are metacentric, 5, 6, 8 and 9 are submetacentric, and 10 and the X chromosome are subacrocentric. The y is a dot.

*H.asperatus* Rey, 1885 (Fig. 9l). The configuration of the karyotype resembles that of *H.strigifrons*, many of the autosomes giving the impression of having very large C-bands.

*H.pumilio* Erichson, 1837 (Fig. 9m, n). Autosomes 1–4 are metacentric, 5–9 are submetacentric, and 10 and the X chromosome are subacrocentric. The X chromosome is about as long as autosome 7, and the almost dot-like y is about a quarter of the length of the X.

*H.croaticus* Kuwert, 1886 (Fig. 9o). Autosomes 1–4 and 6 are metacentric, 5–8 are submetacentric, and 9, 10 and the X chromosome are subacrocentric. The y chromosome is a dot and the X is slightly smaller that autosome 10.

*H.cincticollis* Guillebeau, 1893 (Fig. 9p). Autosomes 1, 3, 4 and 6 are metacentric, 5, 7, 8 and 10 and the X chromosome are submetacentric, and 9 is subacrocentric. The X chromosome is about the same size as autosome 10 and the y is a dot.

#### *Rhopalohelophorus*, the *H.flavipes* Fabricius, 1792 group, and *H.browni* McCorkle, 1970

Fig. 10a–o

The *H.flavipes* group are mainly dark coloured species, lacking yellow margins to the pronotum. *H.flavipes* and *H.obscurus* Mulsant, 1844 are two of the most widely distributed species in Europe.

*H.flavipes* (Fig. 10a–c). Autosomes 1–8 are metacentric, 9 is metacentric to submetacentric, 10 is acrocentric and the X chromosome, about two thirds the length of autosome 10, is subacrocentric. The y is a dot.

*H.obscurus* (Fig. 10d–g). All the autosomes, and the X-chromosome, are clearly biarmed, metacentric (pairs 1–4 and the X chromosome) or metacentric to submetacentric (pairs 5–10). The X chromosome is about as long as pair 10, and the y is a dot.

*H.algiricus* Motschulsky, 1860 (Fig. 10h, i). This species closely resembles *H.obscurus* but differs in minor aedeagal differences and in the smaller larval head. Chromosomally the only clear difference is in autosome 9, which is subacrocentric.

*H.subarcuatus* Rey, 1885 (Fig. 10j, k). Endemic to Corsica and Sardinia, described by Rey from Corsica but very scarce there and much commoner on Sardinia. The karyotype is similar to that of *H.algiricus* but the X chromosome is clearly not metacentric, and autosome pair 9 is more nearly metacentric.

*H.seidlitzi* Kuwert, 1885 (Fig. 10l, m). Endemic to Spain and Portugal where its range overlaps with those of *H.flavipes* and, in the north, *H.obscurus*. The karyotype is very similar to that of *H.flavipes*, the most obvious difference being the subacrocentric autosome 7, which is metacentric in *H.flavipes*.

*H.browni* McCorkle, 1970 ex Angus, 1970b (Fig. 10n, o). This Holarctic species was originally described from tundra in the Canadian Northwest Territories (Mackenzie delta) and Yukon, and Alaska. It is widespread and common in the Baikal area of East Siberia and in central Yakutia and extends to the Russian Far East (Primorye). It is scarce in Mongolia and in China is known from Nei Mongol and Heilongjiang. Angus (2019) refers to variation of the aedeagal strut length in *H.browni*, but further (as yet unpublished) data indicate that this variation is more or less random and continuous, and thus not a concern in attributing the karyotype. Autosomes 1–6 and the X chromosome are metacentric, 7 and 8 are subacrocentric and 9 and 10 are acrocentric. The X chromosome is about the same size as autosome 10. The y is a dot. Autosome 1 is markedly longer than pair 2, while autosomes 2–6 show a smaller and more even decrease in length. C-banding (Fig. 10p) shows centromeric C-bands on all the chromosomes (except the y), those on autosome 1 being particularly small.

### ﻿Triploids and parthenogenesis

Fig. 11a–g

Within the Helophoridae, parthenogenesis was recorded by Angus (1970c) in Canadian *H.orientalis* Motschulsky, 1860, who established its existence by rearing females for two generations in the laboratory. No chromosome data were available. The first chromosomally proven parthenogenesis was by Angus (1992b) who found triploid female *H.brevipalpis* in the Spanish province of León, accompanied by diploids of both sexes. Fig. 11, a shows a karyotype from a parthenogenetic triploid female. The chromosomes match up in triplets without any difficulty, though it may be noted that in triplets 1 and 7 there is a progressive size decrease in the three replicates and triplets 6 and 8 each have one replicate shorter than the other two.

Fig. 11b, c shows a Giemsa-stained and C-banded karyotype from a triploid female taken by Angus at Ligonichio, Regio Emilia, Italy in 2018. Two chromosomes have been lost from this preparation, shown as missing from triplets 2 and 6. The variation in chromosome length within triplets is less than in the Spanish material, but triplets 6 and 9 appear to have one longer replicate, and triplet 7 one shorter one. The apparently shorter replicate in triplet 10 is clearly the result of the short arm not being extended. A triploid nucleus from a single female from Ponte Scipione (Parma Prov., Italy) (Fig. 11d) shows triplet 1 with a similar gradation in replicate length to that shown by the Spanish karyotype shown in Fig. 11a, and triplets 6 and 8 each have one replicate longer than the others.

Fig. 11e, f shows a karyotype from mid gut of a female *H.orientalis* from Mishan, Heilongjiang, China, Giemsa-stained and C-banded. Triplet 1 shows a gradation in replicate lengths, as in Spanish *H.brevipalpis*, but in the other triplets the replicates are more or less equal in length. It is not possible to identify the X chromosome in these *H.orientalis* preparations as in that species males are known only in the American Rockies, and from one locality near Vladivostok in Russia (Angus 1992a).

The question arises is whether these variations in replicate length within triplets result from slight random variation in rates of chromosome condensation through prophase and into metaphase of mitosis, or whether they result from a hybrid origin of these triploids (allotriploidy), which Simon et al. (2003) report as widespread in invertebrates, including, among insects, some Coleoptera, Phasmatodea and Orthoptera. The problem here is finding candidate species which might be involved in hybrid formation. *H.brevipalpis* is intriguing in this context. Angus (1985,Figs 50–56) illustrated variation in the aedeagus size of populations of *H.brevipalpis*, with specimens from northern France (the lectotype, Fig. 50) and Crete (Fig. 51) having relatively smaller aedeagi, while some, including material from the Shetland Islands (Fig. 54, *H.bulbipalpis* Kuwert, lectotype) and Khorasan, Iran (Fig. 55), (now *H.brevipalpislevantinus* Angus, 1988), have them larger. Two of Rey’s names, *H.mixtus* (Fig. 52), with a smaller aedeagus and *H.insignis* (Fig. 53), with a larger one, both refer to material from Provence (southern France). There is thus appreciable variation within *H.brevipalpis*, which might indicate hitherto undetected cryptic species. It is also worth noting that the Spanish León region where triploids were discovered, is on the edge of the species’ range (Millán et al. 2014).

The case of *H.orientalis* is intractable in view of the very limited distributions of bisexual populations (Angus 1992a).

One occurrence which is relevant is the chance occurrence of a triploid embryo among batches of developing eggs obtained from a female *H.aequalis* brought back to the laboratory from St Flour (Cantal), France in 1987. Fig. 11g. shows this karyotype. Triplets 1, 5, 6 and 7 each have one replicate shorter than the others. There appears to be no possibility that this is of hybrid origin, and in particular, there is no other known species of *Helophorus* s. str. with chromosomes sufficiently similar to those of *H.aequalis* to be able to produce such a convincing triploid karyotype. It is worth noting that, in the course of Ph.D. research supervised by Angus, F. Shaarawi obtained a triploid embryo from *Hydrochuselongatus* (Schaller, 1783) (Shaarawi and Angus 1992).

### ﻿Experimental hybrids

Fig. 12a–f

♂ hybrid, *H.lapponicus* ♀ lab-reared from Karasuk X ♂ *H.paraminutus*, from Karasuk (Fig. 12a, b). This cross was originally undertaken with a view to obtaining karyotypes in which the condensation of the chromosomes through prophase into metaphase of mitosis was completely synchronised, to see if any minor differences could be found between the apparently identical karyotypes of the parent species. In fact this was not at all what happened. Both the hybrid karyotypes show serious irregularities in chromosome condensation. Autosome pair 1 shows serious differences in the lengths of the replicates, as does pair 4 in Fig. 12, a, and pair 6 in both karyotypes. The smaller chromosomes perhaps have less scope for showing irregularities, but pair 8 in Fig. 12b and pair 9 in Fig. 12 a both show obvious differences. Angus (1986) suggested that there might be differences in the ease with which paternal chromosomes could incorporate non-histone proteins from a predominantly maternal cytoplasm. But now I am less convinced as by the time the embryos were sufficiently developed for chromosome preparations to be made, the cytoplasm would be of hybrid origin. Some of these hybrids were reared through to adulthood and were apparently able to produce functional meiosis with no failures of chromosomes pairing up during prophase (Fig. 12g).

♂ hybrid, *H.minutus* ♀ lab-reared from Egham, Surrey X ♂ *H.paraminutus*, wild-caught, Austria (Fig. 12), c Giemsa-stained, d phase-contrast. As with the *H.lapponicus* X *paraminutus* cross, there is a mismatch of the replicates of chromosome 1 and no *H.paraminutus* chromosome matches the NOR-bearing *H.minutus* chromosome 4. Angus (1986) shuffled the order of the *H.paraminutus* chromosomes 7–9, to get a better match with those of *H.minutus*. Partly this was a by-product of the reversal of *H.minutus* chromosomes 8 and 9 used in that paper, but with the chromosomes now being arranged on-screen with Photoshop the standard arrangement of the *H.paraminutus* karyotype is used. Chromosomes 7–9 of the two species differ by a pericentric inversion, 7 metacentric in *H.minutus*, acrocentric in *paraminutus*, 8 subacrocentric in *H.minutus*, acrocentric in *paraminutus*, and 9 metacentric in *H.minutus*, acrocentric, possibly with a terminal NOR in the short arm in *paraminutus*. It should at this stage be stressed that this represents the minimum number of differences between homologous chromosomes of the two species. The true extent of the differences may be greater!

♀ Giemsa-stained (Fig. 12e) and ♂ C-banded (Fig. 2f) hybrid embryos, *H.minutus* ♀ lab-reared from Egham, Surrey X ♂ *H.calpensis*, wild-caught, Tarifa, Spain. The Giemsa-stained female karyotype shows no obvious mismatches in autosomes 1–6, with the NOR-bearing chromosome placed as pair 4 in both species. In pair 9 the metacentric *H.minutus* chromosome is longer than the *H.calpensis* submetacentric one. Pairs 7 and 8 show the expected differences in centromere position, but their sizes match quite well. Autosome 10 and the X chromosome both match well. Turning to the incomplete C-banded ♂ karyotype (Fig. 12f), autosome 1 pairs up well, the tip of 1 replicate of autosome 3 lies over another chromosome (perhaps the one shown as *calpensis* pair 9), pairs 4, 5, 8 and 10 are shown as represented by one chromosome each and the longer metacentric *H.calpensis* autosome 9 is suggested to be the one which overlapped something else, possibly the tip of the *H.minutus* autosome 3. The acrocentric and largely heterochromatic y chromosome of *H.calpensis* is clearly recognisable. In general, there is a good overall resemblance in the sequences of sizes of the chromosomes of the two species, but the differences between them may be greater than this suggests.

## ﻿General comments

*Helophorus* chromosomes show useful interspecies variation which is very helpful in delimiting species. They show variation in the size and extent of the C-bands and the distribution of NORs. Where this has been investigated, they show extensive rather fine-grained and fairly uniform chromomeric banding, perhaps equivalent of G-banding. It can be useful in showing where translocations have occurred but this is difficult to demonstrate convincingly, and from the point of view of cytotaxonomy, probably not worth the effort. The obvious polymorphisms encountered result from pericentric inversions, with acrocentric and metacentric versions of the chromosomes involved, and from interpolated heterochromatin (C-bands) into chromosome arms, as in the long variant of the *H.grandis* X chromosome (Fig. 2i, j).

One notable feature of *Helophorus* karyotypes is the frequent occurrence of a particular type of X chromosome–usually not quite the smallest in the nucleus, and subacrocentric to submetacentric. Angus (1989) referred to this as the *H.minutus* pattern of X chromosome. It is shown by some members of the subgenus Helophorus s. str.–*H.thauma*, *aequalis*, *grandis* (short form) (Fig. 2e–i) and *H.hammondi* (Fig. 4c, d). Both species of subgenus Eutrichelophorus have it, *H.micans* (Fig. 4l, m) and *H.oxygonus* (Fig. 4n). In the subgenus Rhopalohelophorus it widespread, occurring in the three *H.leontis* group species (Fig. 6g–j), and *H.nevadensis* (Fig. 6k) in the *Atractohelophorus* group.

Among the other *Rhopalohelophorus* it occurs in *H.nanus* (Fig. 7a–c), *H.pallidipennis* (Fig. 7g, h), *H.minutus* Fig. 8a–c), *H.atlantis* (Fig. 8d, e), *H.calpensis* (Fig. 8f–j), *H.paraminutus* (Fig. 8k, l), *H.lapponicus* (Fig. 8m–p), *H.griseus* (Fig. 9f), *H.jocoteroi* (Fig. 9j, k), *H.strigifrons* (Fig. 9l), *H.asperatus* (Fig. 9m), *H.pumilio* (Fig. 9n, o), *H.croaticus* (Fig. 9p), *H.flavipes* (Fig. 10a–c) (but not in *H.obscurus* and *H.algiricus*, Fig. 10d–i), *H.subarcuatus* (Fig. 10j, k), *H.seidlitzi* (Fig. 10l–n) and *H.browni* (Fig. 10o, p). Other forms of X-chromosomes tend to be metacentric, sometimes a bit larger, but may actually be the longest in the nucleus, as in H. (Gephelophorus) sibiricus (Fig. 4h, i), *H.glacialis* (Fig. 6e, f) and *H.redtenbacheri* (Fig. 7, d).

Chromosome polymorphisms include pericentric inversions, apparently relatively unusual in Coleoptera but present in *Melolonthamelolontha* Linnaeus, 1758 (Scarabaeidae) where one autosome is polymorphic for an inversion, resulting in both metacentric and acrocentric forms. In a secod autosome pair pericentric inversion is suggested as the cause of its departure from the ancestral dinastine metacentric arrangement to its present acrocentric form (Giannoulis et al. 2011).

B-chromosomes may be present, normally small and sometimes difficult to distinguish from the y chromosome, as in *H.nevadensis* (Fig. 6k) and *H.griseus* (Fig. 9g), but may be rather larger, as in *H.jocoteroi* (Fig. 9j).

Parthenogenesis is apparently rare and is currently known in only two species, *H.brevipalpis* (Fig. 11a–d) and *H.orientalis* (Fig. 11e, f), and, as far as is known, is always associated with triploidy.

This contrasts with the situation in *Anacaenalutescens* (Stephens, 1829) (Hydrophilidae) where diploid parthenogenetic females, in populations where males are unknown are always heterozygous for deletion of a small distal portion, beyond a secondary constriction, of autosome pair 8. In some of these populations there are also triploids and these show variation indicating that the triploidy has arisen on separate occasions, after the development of parthenogenesis (Shaarawi and Angus 1991).

The data reported here are summarised in Table 1 which gives the earliest references for material already published. The general karyotype formulae are given for each subgenus and only variations are listed (where they occur) for individual species. The sex chromosomes are listed as Xy. In cases where meiosis is known, it is Xy_p_, the usual polyphagan arrangement, and I have seen nothing to suggest any deviations.
